# Genetic Polymorphisms of a Novel Vascular Susceptibility Gene, Ninjurin2 *(NINJ2)*, Are Associated with a Decreased Risk of Alzheimer's Disease

**DOI:** 10.1371/journal.pone.0020573

**Published:** 2011-06-06

**Authors:** Kun-Pei Lin, Shih-Yuan Chen, Liang-Chuan Lai, Yi-Ling Huang, Jen-Hau Chen, Ta-Fu Chen, Yu Sun, Li-Li Wen, Ping-Keung Yip, Yi-Min Chu, Wei J. Chen, Yen-Ching Chen

**Affiliations:** 1 Institute of Epidemiology and Preventive Medicine, College of Public Health, National Taiwan University, Taipei, Taiwan; 2 Department of Geriatrics and Gerontology, National Taiwan University Hospital, Taipei, Taiwan; 3 Graduate Institute of Physiology, College of Medicine, National Taiwan University, Taipei, Taiwan; 4 Department of Neurology, National Taiwan University Hospital, Taipei, Taiwan; 5 Department of Neurology, En Chu Kong Hospital, Taipei, Taiwan; 6 Department of Laboratory Medicine, En Chu Kong Hospital, Taipei, Taiwan; 7 Center of Neurological Medicine, Cardinal Tien's Hospital, Taipei, Taiwan; 8 Department of Laboratory Medicine, Cardinal Tien's Hospital, Taipei, Taiwan; 9 Department of Public Health, College of Public Health, National Taiwan University, Taipei, Taiwan; 10 Research Center for Genes, Environment, and Human Health, College of Public Health, National Taiwan University, Taipei, Taiwan; University of Valencia, Spain

## Abstract

**Background:**

Accumulated evidences have shown that vascular risk factors, e.g., hypertension, diabetes mellitus and hyperlipidemia, may be related to the risk of dementia. This study investigated the association between genetic polymorphisms of a vascular susceptibility gene, Ninjurin2 *(NINJ2),* and the risk of dementia, which has not been explored previously.

**Methods:**

A total of 275 Alzheimer's disease (AD) patients and 119 vascular dementia (VaD) patients aged 50 or older were recruited from three teaching hospitals from 2007 to 2010. Healthy controls (n = 423) with the same age of cases were recruited from the health checkup and volunteers worked at the hospital during the same time period. Five common (frequency >5%) haplotype-tagging single nucleotide polymorphisms (htSNPs) in *NINJ2* were genotyped to test for the association between sequence variants of *NINJ2* and dementia risk, and how vascular risk factors modify this association.

**Results:**

Homozygosity of two *NINJ2* SNPs was significantly associated with a decreased risk of AD [rs11833579: adjusted odds ratio (AOR) = 0.43; 95% confidence interval (CI)  = 0.23–0.80; rs12425791: AOR  = 0.33, 95% CI  = 0.12–0.96]. Five common haplotypes (cumulative frequency  = 97%) were identified. The global test for the association between *NINJ2* haplotypes and AD was significant (*p* = 0.03). Haplotype CAGGA was significantly associated with a decreased risk of AD (AOR  = 0.32, 95% CI  = 0.11–0.94). No associations were observed for VaD.

**Conclusion:**

Inherited polymorphisms of the vascular susceptibility gene *NINJ2* were associated with AD risk.

## Introduction

Dementia is a common neurodegenerative disorders in the elderly. In the United States, dementia ranked the fifth and eighth leading cause of death in women and men aged 65 years or older in 2003, respectively [1]. In Taiwan, the aging rate ranks 2^nd^ in the world and the prevalence of dementia ranged from 2.5% to 4.4% in the elderly in 1990s [2], [3]. Therefore, dementia is an important health issue in the aging population.

Hypercholesteremia, diabetes mellitus (DM), hypertension, apolipoprotein E (*APOE*) genotype, and atherosclerosis are vascular risk factors involved in chronic inflammation, neurotoxicity and neurodegeneration, which lead to the subsequent cerebral vascular disease, vascular dementia (VaD), and Alzheimer's disease (AD) previously [4], [5], [6], [7]. Limited vascular susceptibility genes were identified in a previous genome-wide association studies (GWAS) for AD risk [8]. A recent GWAS found two single nucleotide polymorphisms (SNPs), rs11833579 and rs12425791, near *NINJ2* were associated with the risk of ischemic stroke [9]. Ischemic stroke and dementia share common mechanisms in pathophysiology, e.g., cerebral hypoperfusion, neurotoxicity, and inflammation [10], [11]. Therefore, it is possible that *NINJ2* plays an important vascular role in AD pathogenesis.

NINJ2 is a transmembrane protein that mediates cell-to-cell and cell-to-extracellular matrix interactions during development, differentiation, and regeneration of the nervous system [12], [13], [14]. The gene encodes NINJ2 is located on chromosome 12p13. It is expressed in brain radial glia cell and lymphocytes [13] and is up-regulated when the nerve is injured. NINJ2 promotes neurite outgrowth and has been related to ischemic tolerance [13]. NINJ2 also interacts with several substances, e.g., L1, N-CAM, and J1 adhesion molecules, which may coordinate a cascade of interactions between leukocyte and endothelial cells and thus plays an important role on the pathogenesis of inflammatory disorder [15], [16]. These mechanisms have been linked to the development of dementia [17]. Therefore, this study was aimed to examine the association between genetic polymorphisms of *NINJ2* and the risk of AD and VaD. In addition, effect modification by vascular risk factors were explored.

## Materials and Methods

### Study Population

This was a case-control study. A total of 394dementia cases were recruited from the neurology clinics of three teaching hospitals in northern Taiwan from November 2007 to July 2010. Healthy controls (n = 443) were recruited from geriatric health checkup and volunteers of the hospital during the same period of time. All participants were aged 50 years or older and were excluded if they had the history of depression, Parkinson's disease, hemorrhagic stroke, cerebral infarction, or brain tumor. In addition, dementia subtypes other than AD and VaD were excluded. After exclusion of participants without blood samples, 275 AD, 119 VaD patients and 423 controls were included for data analysis. This study was approved by the Institutional Review Boards of En Chu Kong Hospital, Cardinal Tien's Hospital, and College of Public Health, National Taiwan University. Written informed consent was obtained from each study participant. The consent from the legal guardian/next of kin was obtained when patients had serious cognitive impairment.

A self-reported questionnaire was administered to collect information on demography, vascular risk factors, lifestyle, and family history of disease. Blood sample was collect in tubes containing sodium EDTA from each participant. After centrifuged, genomic DNA was extracted from buffy coat by using QuickGene-Mini80 system (Fujifilm, Tokyo, Japan) and then stored in a −80°C freezer.

### Dementia Evaluation

At each hospital, one neurologist performed clinical examinations to screen potential dementia cases. Mini-Mental State Examination (MMSE) and Clinical Dementia Rating (CDR) were used to assess their cognitive function. The diagnosis of dementia was evaluated by Diagnostic and Statistical Manual of Mental Disorders (Fourth Edition) [18] criteria. Head images, computed tomography and magnetic resonance imagings, were taken to confirm the subtype of dementia. Diagnosis of probable (typical AD presentation) AD was based on the National Institute of Neurological and Communicative Disorders and Stroke and the Alzheimer's Disease and Related Disorders Association (NINCDS-ADRDA) Alzheimer's Criteria [19].

Diagnosis of VaD was made according to National Institute of Neurological Disorders and Stroke-Association Internationale pour la Recherche et l'Enseignement en Neurosciences (NINDS-AIREN) criteria [20]. Because of different etiology beween large- and small-vessel dementia, only VaD patients with small vessel related stroke (e.g., lacunar infarction and leukoaraiosis) were recruited. The cognitive function of controls was assessed using Short Portable Mental Status Questionnaire [21] to exclude participants with possible dementia and other mental disorders, e.g., Parkinson's disease and meningioma.

### SNP Selection and Genotyping Assay

Common (frequency >0.05) SNPs in *NINJ2* were identified from Han Chinese in Beijing, China (CHB) genotype data of the International HapMap Project (http://hapmap.ncbi.nlm.nih.gov). Haplotype block was defined by Haploview (http://www.broadinstitute.org/haploview/haploview) using the modified Gabriel algorithm [22], [23]. Haplotype-tagging SNPs (htSNPs) were selected from these common SNPs using tagSNP program [24]. SNPs, rs11833579 and rs12425791, associated with stroke risk in a previous GWAS [9] were also included. *APOE* genotypes were determined by the assay developed by Chapman et al. [25] TaqMan Assay (Applied Biosystems Inc., CA, USA) was used to determine genotypes of *NINJ2* htSNPs. Genotyping success rate was greater than 95% for all SNPs. Quality control samples were replicates of 5% study participants and the concordance rate was 100%.

### Statistical Analysis

The Hardy-Weinberg equilibrium (HWE) test was performed to examine possible genotyping error for each SNP among controls. The expectation-maximization algorithm was applied to estimate haplotype frequencies. Logistic regression models were used to estimate adjusted odds ratios (AORs) and 95% confidence intervals (CIs) for dementia (AD or VaD) in participants carrying either one or two versus zero copies of the minor allele of each SNP and each multilocus haplotype. Age, gender, and *APOE e4* status were adjusted in the models as potential confounders. The type I error rate was controlled by false discovery rate (FDR) and the single multiple-degree-of-freedom test (global test) for the association between *NINJ2* SNPs or haplotypes and dementia risk. Given a significant global test, haplotype- and SNP-specific tests can provide some guidance as to which variant(s) contributes to the significant global test.

Vascular risk factors [e.g., body mass index (BMI), smoking history, alcohol consumption, DM, hypertension, hyperlipidemia, cardiovascular disease, *APOE e*4 status] were known risk factors of dementia. This study explored how these factors modified the association between *NINJ2* genotypes and the risk of AD and VaD by using the likelihood ratio test. We also tested the association between *NINJ2* polymorphisms and the risk of AD or VaD stratified by *APOE e*4 status. SAS version 9.1 (SAS Institute, Cary, NC) was used for statistical analyses and all statistical tests were two-sided.

## Results

This study included 275 incident AD cases, 119 small vessel VaD cases, and 423 controls. As compared with controls (Table 1), AD cases were older (78.2 vs. 71.4 years old), included more females (65% vs. 57%), had a lower education level (≤6 years: 51% vs. 10%), more smokers (22% vs. 15%), more DM (18% vs. 12%) and hypertension (39% vs. 49%) history, fewer with hyperlipidemia (18% vs. 29%), and more *APOE e4* carriers (40% vs. 15%). The distributions of BMI, alcohol consumption, and history of cardiovascular disease were similar between AD and controls.

**Table 1:** Characteristics of the study population.

Variables	ADN = 275	VaDN = 119	ControlN = 423
Age (mean±SD)	78.2±8.0*	78.6±7.1*	71.4±7.4
Female (%)	180 (65)*	63 (53)	240 (57)
Education (%)≤6 years6–12years>12 years	139 (51)*95 (35)41 (15)	72 (59)*36 (30)14 (11)	43 (10)176 (42)204 (48)
BMI at age 40 s kg/m^2^(mean±SD)	22.4±3.2	24.1±2.9*	22.0±2.8
Cigarette smoking (%)	59 (22)*	35 (30)*	63 (15)
Alcohol consumption (%)	36 (13)	21 (18)*	42 (10)
DM (%)	49 (18)*	43 (36)*	52 (12)
Hypertension (%)	107 (39)*	78 (66)*	207 (49)
Hyperlipidemia (%)	49 (18)*	28 (24)	122 (29)
Cardiovascular disease (%)	63 (23)	38 (32)	119 (28)
*APOE e*4 carriers (%)	109 (40)*	25 (21)	61 (15)

*p<0.05 for comparing cases (AD and VaD) and controls.

Abbreviations: AD, Alzheimer's disease; VaD, vascular dementia; BMI, body mass index; DM, diabetes mellitus; *APOE* e4, apolipoprotein E e4.

VaD cases were older (78.6 vs. 71.4), had a lower education level (≤6 years: 59% vs. 10%), a higher BMI (24.1 vs. 22 kg/m^2^), more smokers (30% vs. 15%), higher alcohol consumption (18% vs. 10%), and more DM (36% vs. 12%) and hypertension history (66% vs. 49%) as compared with controls (Table 1). The distributions of gender, hyperlipidemia, cardiovascular disease, and *APOE e4* status were similar between VaD cases and controls.

Five htSNPs of *NINJ2* were genotyped. In this study, the minor allele frequencies (MAFs) of the five SNPs ranged from 9% to 43%, which were similar to the MAFs of CHB genotype data in HapMap database. All *NINJ2* SNPs were in HWE among controls (Table 2). For each SNP, the genotype frequencies were not significantly different by disease status (data not shown).

**Table 2:** Characteristics of *NINJ2* haplotype tagging SNPs.

SNP name	Nucleotide change	Location	rs no.	Minor allele frequency (controls)	HWE *p* (controls)	Minor allele frequency (cases)	HWE *p* (cases)
SNP1	C→A	5′UTR	rs4980959	0.39	0.99	0.42	0.23
SNP2	G→A	intron	rs11833579	0.35	0.55	0.30	0.29
SNP3	A→G	intron	rs7298096	0.43	0.79	0.40	0.55
SNP4	G→A	intron	rs7314661	0.09	0.39	0.10	0.36
SNP5	G→A	intron	rs12425791	0.25	0.26	0.21	0.01

Abbreviations: SNP, single nucleotide polymorphism; HWE, Hardy-Weinberg equilibrium; UTR, untranslated region.

Participants carrying two copies of variant SNP2 or SNP5 had a significantly decreased risk of AD (SNP2: AOR  = 0.43, 95% CI  = 0.23–0.80; SNP5: AOR  = 0.33, 95% CI  = 0.12–0.96, Table 3) as compared with non-carriers. SNP2 remained significantly associated with AD risk after controlling for FDR (Table 3). In contrast, no *NINJ2* SNP was associated with VaD (Table 3). After stratified by *APOE e*4 genotype, SNP2 still significantly associated with AD among non-*APOE e*4 carriers after controlling for FDR (AOR  = 0.38, 95% CI  = 0.18–0.82, Table 4), and only *NINJ2* SNP4 was significantly associated with AD risk among *APOE e*4 carriers under additive model (AOR  = 3.03, 95% CI  = 1.07–8.61, Table 4).

**Table 3:** *NINJ2* SNP analysis by genotype for dementia patients and controls.

Co-dominant model									Additive model
SNP	0 copies		1 copy			2 copies			
	Case/control	OR	Case/control	OR (95%CI)	*p*	Case/control	OR (95%CI)	*p*	OR (95%CI)
AD	(Global test *P*<0.0001)								
SNP1	98/156	1.00	123/198	1.18 (0.81–1.72)	0.35	52/63	1.25 (0.76–2.07)	0.35	1.13 (0.89–1.43)
SNP2	127/172	1.00	121/196	0.97 (0.67–1.39)	0.81	21/49	**0.43 (0.23**–**0.80)**	**0.01** *	**0.76 (0.58**–**0.98)**
SNP3	95/134	1.00	133/206	1.00 (0.69–1.47)	0.87	40/75	0.77 (0.46–1.29)	0.25	0.90 (0.70–1.15)
SNP4	224/346	1.00	46/72	1.09 (0.70–1.71)	0.69	4/2	2.99 (0.48–18.15)	0.23	1.20 (0.80–1.80)
SNP5	162/235	1.00	104/164	1.01 (0.71–1.44)	0.98	5/21	**0.33 (0.12**–**0.96)**	**0.04**	0.84 (0.63–1.14)
VaD	(Global test *P* = 0.43)								
SNP1	47/156	1.00	55/198	1.13 (0.69–1.84)	0.55	15/63	0.83 (0.40–1.69)	0.65	0.97 (0.70–1.34)
SNP2	50/172	1.00	50/196	0.95 (0.58–1.54)	0.96	19/49	0.98 (0.49–1.96)	0.95	0.93 (0.67–1.29)
SNP3	39/134	1.00	59/206	1.05 (0.63–1.73)	0.91	17/75	0.92 (0.46–1.83)	0.64	0.98 (0.71–1.37)
SNP4	89/346	1.00	29/72	1.44 (0.83–2.47)	0.19	0/2	NA	NA	1.27 (0.76–2.15)
SNP5	66/235	1.00	47/164	1.04 (0.65–1.66)	0.83	6/21	1.19 (0.43–3.30)	0.72	1.06 (0.72–1.54)

All models were adjusted for age and gender.

Abbreviation: NA, not applicable.

*Result remains significant after controlling for multiple tests by using FDR.

**Table 4:** *NINJ2* SNP analysis by *APOE e4* status for Alzheimer's disease patients and controls.

Co-dominant model									Additive model
SNP	0 copies		1 copy			2 copies			
	Case/Control	OR	Case/ Control	OR (95%CI)	*p*	Case/ Control	OR (95%CI)	*p*	OR (95%CI)
Non-*APOE e*4 carriers									
SNP1	55/134	1.00	80/172	1.32 (0.83–2.08)	0.22	28/48	1.39 (0.75–2.57)	0.29	1.21 (0.90–1.62)
SNP2	76/148	1.00	72/162	0.97 (0.62–1.50)	0.84	12/42	**0.38 (0.18**–**0.82)**	**0.01** *	**0.73 (0.54**–**1.00)**
SNP3	58/106	1.00	79/181	0.86 (0.55–1.36)	0.42	22/63	0.70 (0.37–1.32)	0.22	0.84 (0.62–1.14)
SNP4	135/288	1.00	27/65	0.95 (0.56–1.64)	0.89	2/2	1.92 (0.25–14.81)	0.53	1.03 (0.63–1.67)
SNP5	102/199	1.00	57/137	0.86 (0.56–1.33)	0.51	4/19	0.38 (0.12–1.24)	0.11	0.77 (0.53–1.10)
*APOE e4* carriers									
SNP1	42/22	1.00	42/23	1.30 (0.58–2.93)	0.45	23/14	0.80 (0.30–2.11)	0.72	0.94 (0.57–1.53)
SNP2	51/22	1.00	48/32	0.78 (0.37–1.67)	0.48	9/7	0.47 (0.14–1.67)	0.24	0.72 (0.41–1.26)
SNP3	37/25	1.00	53/24	1.91 (0.83–4.37)	0.20	18/12	0.74 (0.27–2.03)	0.47	0.97 (0.58–1.60)
SNP4	88/55	1.00	19/6	2.73 (0.90–8.25)	0.08	2/0	NA	NA	**3.03 (1.07**–**8.61)**
SNP5	59/33	1.00	47/26	1.17 (0.56–2.45)	0.84	1/2	0.38 (0.03–4.56)	0.40	1.00 (0.51–1.95)

All models were adjusted for age and gender.

Abbreviation: NA, not applicable.

*Result remains significant after controlling for multiple tests by using FDR.

**Figure 1:**
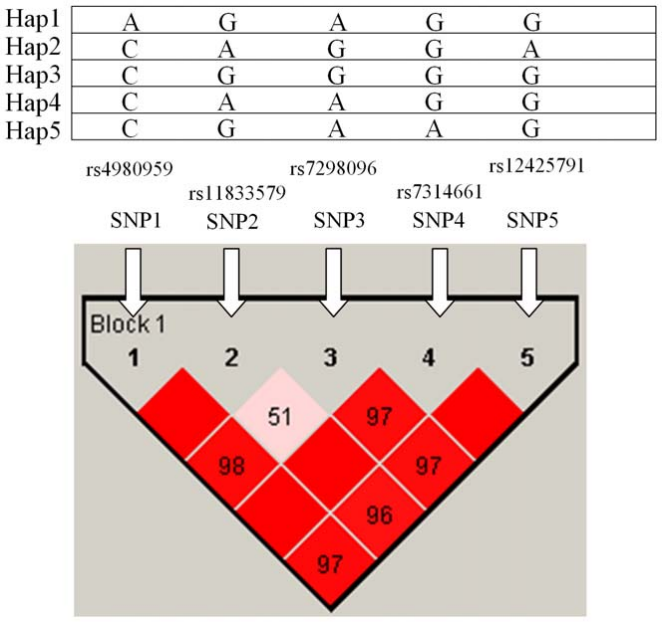
*NINJ2* linkage disequilibrium plot. This plot was generated by Haploview program using the modified Gabriel et al. algorithm using data from this study. Five common haplotype (frequency ≥0.05) were identified and formed one block. The SNP name, e.g., SNP1, SNP2, etc., indicated five htSNP genotyped in this study. The level of pair-wise D', which indicated the degree of linkage disequilibrium between two SNPs, was shown in the linkage disequilibrium structure in red.

Five htSNPs selected from seventeen common (frequency ≥5%) SNPs spanning *NINJ2* formed one block, which was determined by modified Gabriel et al. algorithm [22], [23] (Figure 1). The blocks identified by the default settings in Haploview program were merged if they had the multiallelic D' greater than 0.8 and the cumulative frequency of common (frequency >5%) haplotypes in the merged block was greater than 80%. The five common haplotypes were found with a cumulated frequency of 97.5% in controls (Table 5). The *p* value for global test of the five common haplotypes was 0.03 for AD and 0.70 for VaD. Participants carrying two copies of the minor HAP2 CAGGA had a decreased risk of AD (AOR  = 0.32, 95% CI  = 0.11–0.94). None of *NINJ2* haplotypes was associated with AD and VaD risk under the additive model. Result did not reach statistical significance for VaD.

**Table 5:** ORs between *NINJ2* haplotypes and the risk of dementia.

Co-dominant model										Additive model
Haplotype	Prevalence in controls, %	0 copies		1 copy			2 copies			OR (95%CI)
		Case/Control	OR	Case/Control	OR (95%CI)	*p*	Case/Control	OR (95%CI)	*p*	
AD (Global test *p* = 0.03)										
HAP1: AGAGG	38.0	99/162	1.00	119/196	1.20 (0.82–1.74)	0.35	51/61	1.30 (0.79–2.14)	0.30	1.15 (0.91–1.46)
HAP2: CAGGA	23.8	169/241	1.00	99/161	0.98 (0.69–1.41)	0.93	5/19	**0.32 (0.11**–**0.94)**	**0.04**	0.83 (0.61–1.12)
HAP3: CGGGG	16.8	179/289	1.00	81/114	1.17 (0.80–1.71)	0.42	7/14	0.67 (0.24–1.86)	0.44	1.03 (0.75–1.42)
HAP4: CAAGG	10.0	221/335	1.00	43/70	0.83 (0.53–1.31)	0.43	2/6	0.21 (0.04–1.18)	0.08	0.72 (0.48–1.08)
HAP5: CGAAG	8.9	225/348	1.00	45/71	1.08 (0.69–1.70)	0.74	4/2	2.99 (0.49–18.21)	0.23	1.19 (0.79–1.78)
VaD (Global test *p* = 0.70)										
HAP1: AGGGG	38.0	45/162	1.00	54/198	1.19 (0.73–1.95)	0.51	15/61	0.91 (0.45–1.87)	0.82	1.01 (0.73–1.40)
HAP2: CAAGA	23.8	64/240	1.00	45/162	1.04 (0.65–1.67)	0.84	6/19	1.31 (0.46–3.70)	0.59	1.09 (0.74–1.59)
HAP3: CGAGG	16.8	81/291	1.00	32/113	1.17 (0.71–1.95)	0.60	1/14	0.21 (0.03–1.76)	0.15	0.92 (0.60–1.42)
HAP4: CAGGG	10.0	93/337	1.00	17/70	0.69 (0.37–1.32)	0.25	3/6	0.78 (0.17–3.69)	0.77	0.76 (0.46–1.27)
HAP5: CGGAG	8.9	86/348	1.00	28/71	1.42 (0.82–2.45)	0.20	0/2	NA	NA	1.27 (0.75–2.15)
Cumulative frequency	97.5									

Abbreviation: NA, not applicable.

All models were adjusted for age and gender.

Among the vascular risk factors explored in this study (e.g., hypertension, DM, hyperlipidemia, smoking, and high BMI), hyperlipidemia is the only one significantly modify the association between *NINJ2* polymorphisms and the risk of AD. Hyperlipidemia status significantly lower the risk of AD (AOR  = 0.43, 95% CI  = 0.27–0.71) as compared with participants without this condition. Hyperlipidemia significantly modified the association between HAP2 CAGGA and AD risk under the recessive model (*p_interaction_*  = 0.02, Table 6). After stratification by hyperlipidemia status, participants without hyperlipidemia and carrying two copies of minor HAP2 had a decreased risk of AD as compared to those carrying 0 or 1 copy of HAP2 (AOR  = 0.08, 95% CI  = 0.01–0.71, Table 6). In contrast, increased AD risk was observed in participants with hyperlipidemia and carrying 2 copies of minor HAP2 (AOR  = 1.40, 95% CI  = 0.28–7.02). Other vascular risk factors did not modify the relationship between *NINJ2* haplotypes and AD risk (data not shown). No significant interactions were observed for VaD risk (data not shown).

**Table 6:** Interaction between HAP2 and hyperlipidemia on AD risk.

Hyperlipidemia	HAP2 (CAGGA)				*p*_interaction_ *
	0 or 1 copy		2 copies		
	Case/Control	OR	Case/Control	OR (95%CI)	
No	221/285	1.00	1/15	**0.08 (0.01**–**0.71)**	**0.02**
Yes	45/117	1.00	4/5	1.40 (0.28–7.02)	

All models were adjusted for age, gender, and *APOE e4*.

*p value was obtained by using the recessive model.

## Discussion

**Figure 2:**
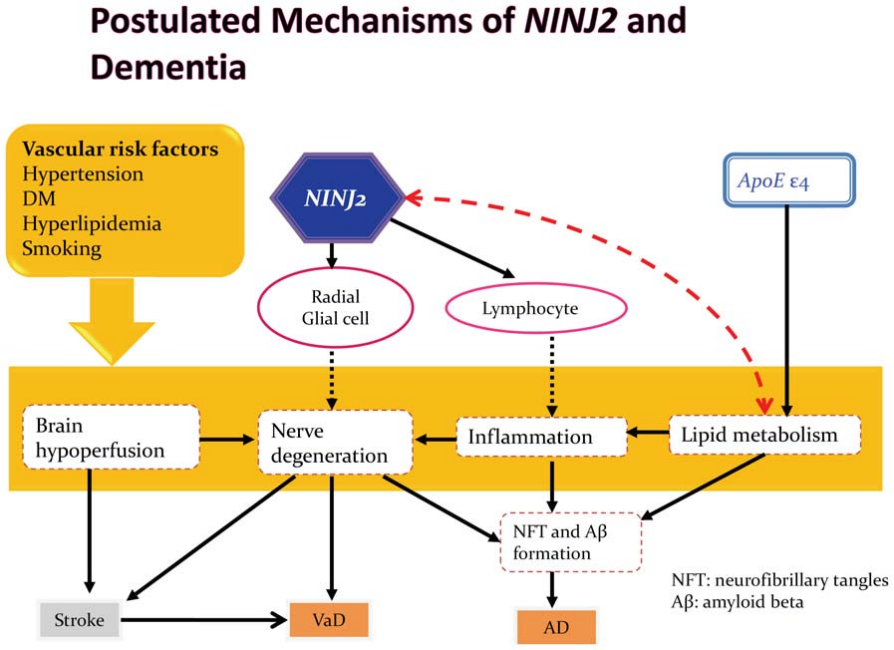
Postulated pathway of *NINJ2* and mediators involved in the formation of dementia. Solid lines indicated pathways have been well documented; dotted lines indicated speculated pathway. NFT denotes neurofibrillar tangle. Aβ denotes beta amyloid.

This study found that *NINJ2* rs11833579 (SNP2), rs12425791 (SNP5) and HAP2 (CAGGA) were significantly associated with a decreased risk of AD, which has not been reported previously. These two SNPs were related to ischemic stroke in a previous GWAS [9]. This indicated that dementia and ischemic stroke may share common risk factors, e.g., vascular risk factors [26], [27] and cerebrovascular disease [28], [29]. Neurofibrillar tangle (NFT) and beta amyloid are important markers in the brain of AD patients and they are also markers for the neurotoxic cascades and degeneration process. NINJ2 plays a role in neurite growth, ischemic tolerance, and inflammation response, its sequence variations may reduce or block the signaling of the immune response and thus lead to the formation of NFT and beta amyloid and subsequently lower the risk of AD (Figure 2). NINJ2 also affects dementia risk via nerve regeneration. A research found that the expression level of NINJ2 was associated with axonal regeneration [30], which may explain how the brain tolerates ischemic insults.

Participants carrying two copy of minor HAP2 had a 0.32-fold decreased risk of AD (Table 5). SNP2, SNP3 and SNP5 were SNPs carrying variant alleles in HAP2. Because each variant allele was associated with decreased AD risk (although SNP3 did not reach statistical significance), HAP2 may result in a protective effect on AD. However, no SNP or haplotype was related to small-vessel VaD in this study, probably due to small sample size (119 VaD cases) and moderate genetic effect. Our findings suggested that *NINJ2* polymorphisms played an important role in dementia risk, which has not been explored so far.

It is well established that APOE plays a role in the metabolism of cholesterol, which regulates the formation of neurofibrillar tangle and Aβ and the subsequent AD risk. After stratification by *APOE e*4 status, variant SNP2 became a stronger protector for AD among non-*APOE e*4 carriers (AOR  = 0.38, 95% CI  = 0.18–0.82) as compared to *APOE e*4 carriers (AOR  = 0.47, 95% CI  = 0.14–1.67). This may be attributable to the joint effect of lack of *APOE e4* allele and *NINJ2* SNP2.

Vascular risk factors (e.g., hypertension, DM, smoking, and higher BMI), have been associated with VaD risk in previous studies [27], [31]. We found that history of hyperlipidemia and hypertension were protective factors for AD (data not shown), which was consistent with a recent cohort study [32]. First, this may be a result of using of medication for cardiovascular disease, e.g., statin [33] and angiotensin converting enzyme inhibitors [34]. It is also possible that the participants recruited in this study were survivors of people with high-risk of vascular diseases. Therefore, they may carry genes related to lower risk of hyperlipidemia or hypertension. In addition, we found that hyperlipidemia significantly modified the association between HAP2 and AD risk. It is possible that hyperlipidemia was associated with the change of acetylcholine, a neurotransmitter in central nervous system, which increases brain perfusion and plays the vascular role in the cholinergic neural system [35]. That is, ischemic insult resulting from vascular risk factor, e.g., hyperlipidemia, may lead to dementia occurrence [36]. However, the underlying mechanism remains to be elucidated.

This study has several strengths. First, the association between *NINJ2* polymorphisms and dementia risk has not been explored previously. Second, the selection of a set of representative htSNPs captured the majority of genetic information of *NINJ2* (r^2^ = 0.79). Third, as compared to SNPs, haplotypes provided a stronger statistical power to detect the association between *NINJ2* sequence variants and dementia because these htSNPs are in highly linkage disequilibrium. In addition, all dementia cases were confirmed by brain imaging to minimize possible misclassification of dementia subtypes. Last, because the clinical presentation of large-vessel ischemia insults varied tremendously, inclusion of patients with small-vessel dementia (e.g., lacunar infarction and leukoaraiosis) provided homogeneous outcome.

This study had some limitations as well. First, the information of vascular risk factors (e.g., hypertension, DM, and hyperlipidemia) was obtained from a self-report questionnaire instead of medical charts. However, these diseases are major health issues, and participants' awareness of these diseases/conditions was inquired in the questionnaire if their disease/condition was diagnosed by physicians. Therefore, information bias should not be a concern. Second, medications for treating cardiovascular or cerebrovascular diseases may affect the AD course [37]. This information may not be available because our participants were recruited from neurology clinics and they tended to look for other physicians for diagnosis and treatment of vascular diseases. Last, this study included 122 VaD cases and may not have sufficient statistical power to assess the associations between *NINJ2* polymorphisms and VaD risk.

*NINJ2* SNPs and a haplotype were significantly associated with AD risk, which has not been explored previously, possibly through inflammation and metabolic pathways. Also, we found that hyperlipidemia significantly modify this association. Future large studies are warranted to explore these associations.
